# Soap and Soap Making

**Published:** 1882-12

**Authors:** 


### SOAP AND SOAP MAKING.

IT is a remarkable fact that among all the industries developed by the wants of mankind, none of equal importance has made so little progress as soap making. Pliny informs us that the Gauls were the inventors of soap made of tallow and wood ashes: a soap-boiling shop has been discovered in Pompeii. It is certain that from the beginning of the Christian era, until a period within the memory of men now living, there was absolutely no progress; and to this present time so little has been done to improve the quality of soap, and at the same time to economize time and materials, that modern soap factories seem like relics of the "dead past."

We did not intend to write an article on soap making at this time, as we were awaiting the development of experiments now being made, which promise most important results, but we do not believe in condemning an article or a process unless we have something better to offer, and thus we will briefly state our reasons for desiring radical improvements in the process of soap making.

The ordinary soap of commerce is made by boiling grease and alkali in an open kettle; a portion of the alkali combines with a portion of the grease and forms soap, while the weaker part, called "spent lye" is drawn off, carrying the *glycerine* with it. Large quantities of salt are also used to bring about the result. More alkali is then added, and after boiling about 4 days the soap is said to be made; but in fact about 20 per cent. of the grease remains unchanged. The result is that from 100 pounds of grease about 130 pounds of soap is made; the quantity is increased more or less by the addition of rosin and other adulterations. If impure grease is used, as from diseased animals, that portion not converted into soap retains its poisonous properties, and no doubt is the cause of many diseases whose origin seems involved in mystery.

By the above process all the glycerine is lost, amounting to 12 per cent.; a quantity of salt is wasted, and an inferior soap is produced.

The experiments above referred to as now in progress, have beyond question, already accomplished the following results:

1st. The complete saponification of the grease, so that any germs of disease that may exist are totally destroyed.

2d. The saving of all the glycerine contained in the grease which is incorporated with the soap, improving its quality.

3d. Increase of quantity, since all the material used is converted into soap, thus giving 190 pounds of soap for every 100 pounds of grease used, instead of 130, as by the old process.

4th. Saving the value of the salt, as none is used in the new process.

5th. The ability to convert cotton seed and other vegetable oils into superior soap.

6th. The saving of expense, since the cost of machinery for manufacturing soap by the new process is not one-quarter as much as by the old.

7th. Saving of time; a batch requiring 4 days by the old process, can be made in a day by the new.

8th. Superior quality, as the new process soaps are firmer, shrink less and will do more washing than any made by the old process.

9th. Saving of fuel and labor.

We shall have occasion to refer to the subject again as experiments progress. We vouch for what has been written, since these experiments were made under our own observation.

